# Prevention of Colitis-Associated Cancer via Oral Administration of M13-Loaded Lipid Nanoparticles

**DOI:** 10.3390/pharmaceutics15092331

**Published:** 2023-09-16

**Authors:** Dingpei Long, Zahra Alghoul, Junsik Sung, Chunhua Yang, Didier Merlin

**Affiliations:** 1 Digestive Disease Research Group, Institute for Biomedical Sciences, Georgia State University, Atlanta, GA 30303, USA; dlong26@gsu.edu (D.L.); zalghoul@gsu.edu (Z.A.); jsung9@gsu.edu (J.S.); dmerlin@gsu.edu (D.M.); 2 Department of Chemistry, Georgia State University, Atlanta, GA 30303, USA; 3 Gastroenterology Research, Atlanta Veterans Affairs Medical Center, Decatur, GA 30302, USA

**Keywords:** M13, nano-liposome, IL-10 knockout, chronic inflammation-induced cancer, ulcerative colitis (UC)

## Abstract

Inflammatory bowel disease (IBD), which includes ulcerative colitis (UC) and Crohn’s disease, is known to increase the risk of colitis-associated cancer (CAC). CAC has been found to be unresponsive to standard chemotherapy regimens, and the current treatments do not utilize effective small-molecule drugs and colon-targeted delivery systems. Previous studies indicated that the M13–nano-liposome (NL) formulation can effectively target the colon and reshape the gut microbiota in ex vivo cultures, generating altered microbial metabolites that can efficiently prevent chronic UC. In this study, we tested the cancer cell uptake ability of the NL formulation and investigated the potential of the M13–NL formulation to prevent CAC in the azoxymethane (AOM)-exposed IL10^−/−^ mouse model. Our findings demonstrate that oral administration of M13–NL prevents tumor development in AOM-exposed IL10^−/−^ mice, suggesting that M13–NL is a promising oral drug formulation for preventing CAC.

## 1. Introduction

Patients with inflammatory bowel disease (IBD), which includes ulcerative colitis (UC) and Crohn’s disease, have an increased risk of developing colitis-associated cancer (CAC) [1,2]. The early diagnosis and treatment of CAC can significantly increase the patient survival rate, because drug treatments are most effective in the early stages of cancer progression [3,4]. Medications such as 5-aminosalicylates (5-ASA), nonsteroidal anti-inflammatory drugs (NSAIDs), corticosteroids, and immunosuppressants may be used to reduce inflammation and lower the risk of CAC in patients with IBD [5,6]. A meta-analysis of observational studies found that the use of 5-ASA was associated with a reduced risk of CAC in patients with IBD [7].

Despite their potential to prevent CAC, the current IBD drug treatments are accompanied by severe side effects [8]. These drugs often require repeated delivery via parenteral routes, and their long-term application is associated with increased risks of heart attack, stroke, and kidney problems [9,10]. For example, the long-term use of NSAIDs can reduce blood flow to the kidneys and cause kidney damage, especially in people with pre-existing kidney disease [11]. In addition, parenteral routes such as intravenous, subcutaneous, and intramuscular injections are not convenient for patients [12]. There is an unmet need for a safe and convenient formulation that can effectively target and release drugs to diseased tissue, reduce systemic side effects, and improve IBD symptoms.

Our previous studies demonstrated that lipids extracted from ginger-derived nanoparticles can be assembled to target specific parts of the digestive tract and support the efficient oral delivery of small molecules and siRNAs [13]. The assembled lipid nanoparticles also trigger less toxicity than traditional polymeric nanoparticles do [14]. Here, we loaded these lipid nanoparticles with M13, which is a promising anti-UC compound [15], and used the generated M13–nano-liposome (NL) as an oral formulation to treat CAC. M13 is a phase II metabolite of the anti-inflammatory natural product, 6-shogaol, that exhibits better tolerability than 6-shogaol do [15]. Structurally, M13 is an amphiphilic compound; this makes it an excellent candidate for being loaded into NL with high efficiency [16].

To test the CAC-preventing efficacy of this formulation, we adopted the AOM-exposed IL10^−/−^ mouse model, which is a preclinical tool commonly used to investigate the development of CAC [17]. This model combines two critical factors involved in CAC development: chronic inflammation and DNA damage. The IL10^−/−^ mouse does not produce the anti-inflammatory cytokine, IL-10, in the colon, and thus experiences chronic inflammation; the compound AOM induces DNA damage in the colon, leading to the development of colorectal tumors in IL-10-null mice [18]. Using this model, we herein assessed the in vivo efficacy of the long-term oral administration of M13–NL against the development of CAC.

## 2. Materials and Methods

### 2.1. Chemicals

6-shogaol (98+% purity) was purchased from Chengdu Alfa Biotechnology Co., Ltd. (Chengdu, China). Reduced L-Glutathione, NaHCO_3_, ethanol (200 proof), methanol, and dichloromethane were purchased from Sigma-Aldrich (St. Louis, MO, USA). Formic acid (98+%, LC-MS grade), phosphate-buffered saline (Corning TM PBS (Corning, NY, USA), 1×), and DiL Stain (1,1′-Dioctadecyl-3,3,3′,3′-Tetramethylindocarbocyanine Perchlorate (DiIC18(3)) were obtained from Fisher Scientific (Hampton, NH, USA). Fetal bovine serum (FBS) was obtained from Atlanta Biologicals (R&D Flowery Branch, GA, USA). Ultrapure deionized water was supplied by a Milli-Q water system (Millipore, Bedford, MA, USA). M13 was synthesized and purified using a method that has been previously published [19].

### 2.2. Fabrication of Nano-Liposomes (NL)

Ginger-derived nanoparticles band 2 (GDNPs-2) were prepared from the fresh ginger rhizome (*Zingiber officinale* Rosc.) using the glucose gradient ultracentrifugation method [14]. Total lipids were extracted from GDNPs-2 using a liquid–liquid extraction method. The assembly of M13–NL was carried out using a thin-film hydration method [16]. In general, a thin lipid–M13 complex film was obtained by mixing 1 mL of M13 (5.0 mg/mL in ethanol) with 1 mL of total GDNPs-2 lipids (5.0 mg/mL in dichloromethane) and drying under reduced pressure (55 °C, 50 Torr). Then, the dried film was suspended in 5 mL of PBS buffer. After being bath-sonicated for ~5 min, an equal volume of PBS buffer was added and sonicated for another 5 min. Empty NL (as control) was made by using the identical protocol without the addition of M13, as described above. For the in vitro cellular uptake experiment, NL was labeled with a lipophilic carbocyanine dye (DiL, D282, Fisher Scientific, Waltham, MA, USA) to form DiL-labeled NL (DiL–NL), as has been previously described [20].

### 2.3. Characterization of NL

A scanning electron microscope (SEM) was used to visualize M13–NL, NL, and DiL–NL. In general, sample suspensions were dried for 3 h at room temperature, glued to holders, and coated with an approximately 15 nm layer of gold using a Denton Vacuum Desk II sputter coater (Moorestown, NJ, USA). Then, the processed samples were imaged at 15 kV by using a Tescan VEGA 3 scanning electron microscope (Tescan Analytics, Fuveau, France) located at the Imaging Core Facility, Georgia State University (Atlanta, GA, USA). For size and zeta-potential characterization, NLs were measured at room temperature using Malvern Zetasiser Nano ZS90 Apparatus (Malvern Instruments, Malvern, UK). 

### 2.4. Cell Uptake Study

Caco-2 BBE epithelial cells were seeded overnight in Falcon^®^ Culture Slides (Corning, Glendale, AZ, USA) with a concentration of 0.5 × 10^5^ per well. Next, 50 μL of DiL-labeled NL was added to 0.5 mL of culture medium and incubated with cells for 0, 12, 24, and 48 h. Negative control cells were set as treated for 0 h. The cells were washed with PBS three times, fixed with 4% paraformaldehyde for 15 min, and permeabilized with 0.1% Triton X-100 for 5 min. Then, the cells were rewashed with PBS and treated with 100 μL of Fluorescein isothiocyanate (FITC)-labeled phalloidin (1:50 dilution in PBS) for 30 min. The cells were washed with PBS twice, dried in the dark, and coverslip-mounted with a mounting medium containing 4-,6-diamidino-2-phenylindole (DAPI). Finally, images were captured using an Olympus fluorescence microscope (Tokyo, Japan) equipped with Hamamatsu Digital Camera ORCA-03G (Hamamatsu Photonics, Shizuoka, Japan).

To compare cellular uptake efficiency, Caco-2 cells were seeded in six-well plates with a concentration of 1 × 10^5^/well and incubated overnight. Cells were then treated with DiL-labeled NLs (2 mg/mL) or blank NLs (control) for different time periods (0, 12, 24, and 48 h). The cells were rinsed with cooled PBS three times and harvested for flow cytometry analysis using an LSRFortessaTM cell analyzer (BD, Franklin Lakes, NJ, USA).

### 2.5. In Vivo Cancer Prevention Test on AOM-Exposed IL10^−/−^ Mouse Model of CAC

#### 2.5.1. Animal

Female IL10^−/−^ mice (B6. 129P2-IL10 tm1Cgn, The Jackson Laboratory, Farmington, CT, USA) were used to establish an orthotopic CAC mouse model using a casting method described previously [21,22]. Mice were housed in an animal facility maintained at 22 ± 2 °C and 30–70% relative humidity with a 12 h light/dark cycle. Diet and water were supplied ad libitum. The IL10^−/−^ mice (12 weeks old) were randomly allocated to three groups (*n* = 7) and intraperitoneal injections of AOM (10 mg/kg) were taken once a week for 6 weeks. After the initial injection of AOM, there were 1, 2, and 2 deaths in the AOM/PBS, AOM/NL, and AOM/M13–NL groups, respectively. Consequently, the number of mice in the AOM/PBS, AOM/NL, and AOM/M13–NL groups was adjusted to 6, 5, and 5 mice, respectively, for the subsequent study. All animal experiments were performed following ARRIVE guidelines 2.0 and with approval from the Georgia State University Institutional Animal Care and Use Committee (IACUC, Protocol # A20039).

#### 2.5.2. Treatment Groups

IL10^−/−^ mice from each group received different treatments from the age of 12 weeks to 31 weeks. Group 1 was the AOM/PBS control (AOM treated with PBS as drinking water), group 2 received the AOM/NL treatment (AOM-treated with 0.0025% (*m*/*v*) NL supplemented in PBS), and group 3 received the AOM/M13–NL treatment (AOM-treated with M13–NL-supplemented (0.0025% M13 loaded into 0.0025% NL) in PBS). Water consumption was measured daily and the dosage orally delivered was calculated in accordance with the drug concentration multiplied by the consumed volume. Additionally, the mice in the PBS control group received an equivalent volume of PBS daily.

#### 2.5.3. Bodyweight, Fecal Lipocalin Measurement, and Colonoscopy

Body weights were measured every two weeks during the study. Pre-weighted frozen fecal samples (~100 mg) from 5-, 9-, 11-, 12-, 17-, 21-, 25- and 29-week-old IL10^−/−^ mice were collected and the levels of fecal lipocalin 2 (Lcn-2) were evaluated using a Duoset mouse Lcn-2 ELISA kit (R&D Systems, Minneapolis, MN, USA) using the method described previously [15]. The 23-, 26-, and 30-week-old IL10^−/−^ mice fasted for 8 h, and then the mice were put under anesthesia with 3% isoflurane, and a direct visualization of the colon was performed using a colonoscope (Storz Veterinary Endoscopy, Goleta, CA, USA) as previously described [23].

#### 2.5.4. Intestinal Microbiota

Feces were collected from IL10^−/−^ mice before the AOM injection (on week 5, named IL10^−/−^ group) and before the sacrifice (on week 31) of each group, respectively, in the therapeutic experiments as described above. Then, fecal samples of the IL10^−/−^, AOM/PBS, AOM/NL, and AOM/M13–NL groups were collected using a sample collection kit containing barcoded tubes provided by Transnetyx (Cordova, TN, USA). In general, fecal samples (2 feces/mouse) were placed in individual tubes containing DNA stabilization buffer and shipped for DNA extraction, library preparation, and sequencing by Transnetyx. Shallow shotgun whole-genome sequencing (WGS) for microbiota analysis was performed by Transnetyx with classification performed by One Codex (https://app.onecodex.com/ (accessed on 5 June 2022); San Francisco, CA, USA). Data analysis (the number and relative abundance of taxa, α-diversity, and β-diversity) was performed on the network cloud platform of One Codex using the Compare Analyses server (https://app.onecodex.com/custom-plots (accessed on 10 June 2022). The number and relative abundance of taxa in some rank (e.g., species or operational taxonomic units (OTUs)) were analyzed and compared using the bar graph, heatmap, pie graph, and Venn diagram. The α-diversity of the samples was measured via the observed species based on OTUs, the Shannon index, and the Simpson index. The β-diversity of the samples was measured via principal component analysis (PCA) derived from Weighted UniFrac and Bray–Curtis distances among different groups.

#### 2.5.5. Tumor Counting, Colon Length, and Spleen Weight

Mice were euthanized at the age of 31 weeks and the lengths of the colons were measured. The colons were cut open longitudinally and the colon tumor number and size were quantified. Meanwhile, other organs (liver, spleen, kidney, and small intestine) were immediately collected after euthanasia. The spleen weights were measured and all the organs were rinsed using PBS (pH 7.4) to remove blood and residual contents.

### 2.6. Intratumoral Microbiota Analysis

In addition, the intestinal tumors of mice from the AOM/PBS, AOM/NL, and AOM/M13–NL groups were separated using a scalpel, and then were placed in individual tubes containing DNA stabilization buffer and shipped for DNA extraction, library preparation, and sequencing by Transnetyx. The WGS of extracted DNA from these tumors and microbiota analysis were also performed via the One Codex platform in accordance with the above method.

### 2.7. Statistical Analysis

GraphPad Prism 9 and Microsoft Excel 2013 were utilized for graphing and data analysis. All the data presented are biological replicates, and the outliers were calculated and removed using the outlier calculator in GraphPad with an alpha value of 0.05. Significance was determined using either an unpaired two-tailed Student’s *t*-test or one-way ANOVA and differences were noted as significant (* *p* < 0.05, ** *p* < 0.01, *** *p* < 0.001, and **** *p* < 0.0001).

## 3. Results

### 3.1. Assembly and Characterization of M13–NL, NL, and DiL-Labeled NL

**Figure 1:**
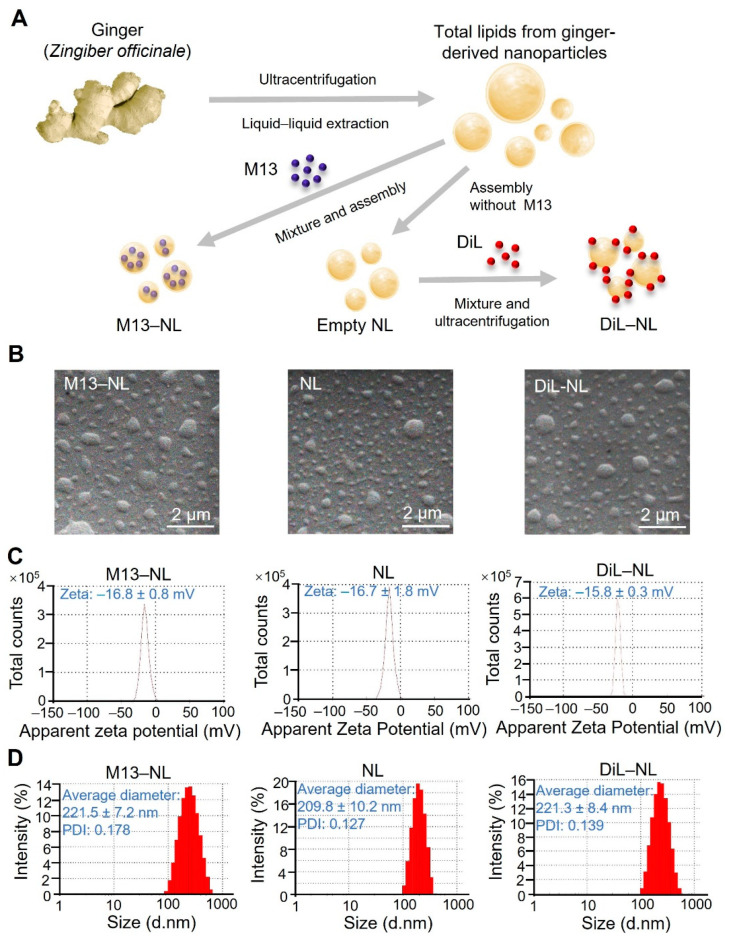
Fabrication and characterization of different types of NL from ginger-derived lipids. (**A**) Schematic illustration of the fabrication of M13–NL, empty NL, and DiL–NL. (**B**) SEM images, (**C**) zeta potentials, and (**D**) size distributions of the different types of NL.

The assembly of NL demonstrated excellent reproducibility across M13-loaded NL, empty NL, and DiL-labeled NL, as shown in Figure 1. A schematic graph illustrates the process of lipid extraction from ginger-derived nanoparticles, the assembly of M13 nano-liposomes, and the labeling of nano-liposomes with DiL (Figure 1A). All three types of nanoparticles were spherical in shape, as observed via SEM (Figure 1B), and had slightly negative surface charges (Figure 1C). Empty NL had a hydrated size of approximately 209.8 nm and a polydispersity index (PDI) of 0.127, while M13–NL had a slightly larger hydrated size of 221.5 nm and a PDI of 0.178 (Figure 1D). DiL-labeled NL had a hydrated size close to that of M13–NL, at ~221.3 nm (Figure 1D). These similar characteristics, including size, PDI (the polydispersity index), and zeta potential, indicate that NL is a robust platform capable of accommodating drug loading and dye labeling. Furthermore, the low PDI values (<0.2) of these nanoparticles indicate a highly uniform particle population with minimal size variation [24]. The stability evaluation of NL and M13–NL revealed that these particles retained their size and PDI for a period of 7 days, as illustrated in Supplementary Figure S1. 

### 3.2. NL Is Continuously Internalized by Cancerous Epithelial Cells

**Figure 2:**
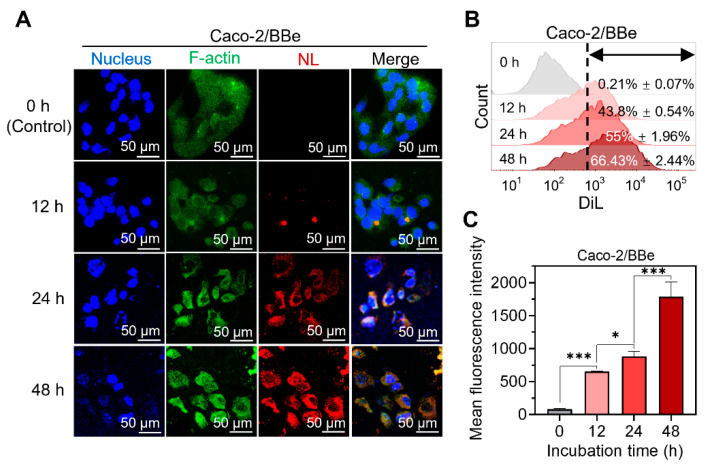
Profiles of the cellular uptake of DiL–NL by Caco-2/Bbe cells. (**A**) Fluorescence images showing the cellular uptake of DiL–NL (10 μg/mL; a total of 1 μM DiL) by Caco-2/Bbe cells for 0 (control), 12, 24, or 48 h. (**B**) Flow cytometry histogram profiles of fluorescence intensity for Caco-2/Bbe cells treated with DiL–NL (10 μg/mL) for 0, 12, 24, or 48 h. (**C**) The quantification of DiL fluorescence intensity in Caco-2/Bbe cells treated with DiL–NL (10 μg/mL) for 0, 12, 24, or 48 h. Error bars represent one standard deviation (*n* = 3; * *p* < 0.05 and *** *p* < 0.001).

To demonstrate the effectiveness of NL delivery, we conducted an in vitro study using Caco-2/BBe cells to evaluate the cellular uptake of NL over an extended treatment period (up to 48 h). As depicted in Figure 2A, cells incubated with DiL-labeled NL exhibited noticeable red fluorescence signals after 24 h, whereas control cells incubated without DiL-labeled NL did not. The red fluorescence intensity of DiL–NL increased significantly with the incubation time (12, 24, and 48 h), indicating that there was a continued increase in the amount of DiL–NL internalized by the Caco-2/Bbe cells (Figure 2A). The cellular uptake efficiency of DiL–NL was quantified via flow cytometry, which confirmed that the cells exhibited higher NL uptake as the incubation time was extended to 48 h (Figure 2B,C). These findings suggest that NL can be continuously and efficiently taken up by and accumulated within Caco-2/Bbe cells for at least 48 h.

### 3.3. Oral Administration of M13–NL Prevents Colonic Tumorigenesis in AOM-Exposed IL10^−/−^ Mice

**Figure 3:**
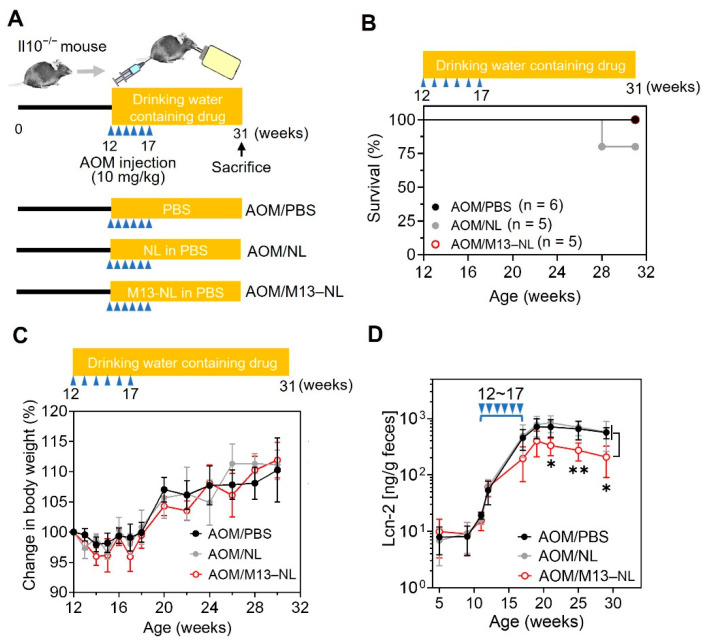
Establishment of AOM-exposed IL10^−/−^ mouse model. (**A**) Timeline for AOM-exposed CAC tumorigenesis in IL10^−/−^ mice. PBS, NL (25 μg/mL), or M13–NL (25 μg/mL M13 loaded into 25 μg/mL NL) were given in drinking water from weeks 12 to 31. (**B**) Survival rates of AOM-exposed IL10^−/−^ mice from each group. Kaplan–Meier curves were generated using GraphPad Prism software 8.0. (**C**) Changes in body weights of AOM-exposed IL10^−/−^ mice from each group throughout the experiment. Mouse body weight over time, normalized as a percentage of the 12-week-old body weight and given as the mean of each group (*n* = 5 or 6). (**D**) Lcn-2 levels of fecal samples obtained from weeks 5 to 29 (*n* = 5). Blue triangles indicate the AOM injection schedule. * *p* < 0.05 and ** *p* < 0.01.

Next, the AOM-exposed IL10^−/−^ mouse model was used to evaluate the ability of the long-term oral administration of M13–NL to prevent colon tumorigenesis. As shown in Figure 3A, 12-week-old IL10^−/−^ female mice were randomly allocated to three groups (*n* = 5 or 6 per group) and orally administered PBS (AOM/PBS group), PBS with NL (0.0025% (*m*/*v*); AOM/NL group), or PBS with M13–NL (0.0025% M13 (*m*/*v*) loaded into 0.0025% NL; AOM/M13–NL group) via their drinking water from the age of 12 to 31 weeks. Also beginning at 12 weeks of age, the mice were given intraperitoneal injections of AOM (10 mg/kg) once a week for 6 weeks. We first determined the effect of oral PBS, NL, or M13–NL on the survival and body weight of AOM-exposed IL10^−/−^ mice throughout the experimental period. No mortality was observed in most of the groups during the study (Figure 3B); one mouse in the AOM/NL group was found dead at week 28. Weeks refer to total weeks of age. We observed that the body weights of the mice in the three groups decreased slightly during the AOM injection (12 to 17 weeks) and gradually increased thereafter until sacrifice, and there was no significant difference in the body weight changes of mice among the three groups (Figure 3C). These results suggest that long-term M13–NL treatment may not induce severe systemic toxicity in these mice. Next, we assessed the anti-inflammatory effect of long-term M13–NL treatment by detecting fecal lipocalin 2 (Lcn-2), which is a non-invasive biomarker for monitoring intestinal inflammation in mice [25]. We observed that the levels of fecal Lcn-2 gradually increased in all groups from weeks 11 to 19 (Figure 3D). Unlike the AOM/PBS and AOM/NL groups, the AOM/M13–NL group showed a downward trend in the fecal Lcn-2 concentration beginning at week 17 and significantly lower levels of fecal Lcn-2 from week 21 to the end of the experiment (Figure 3D).

**Figure 4:**
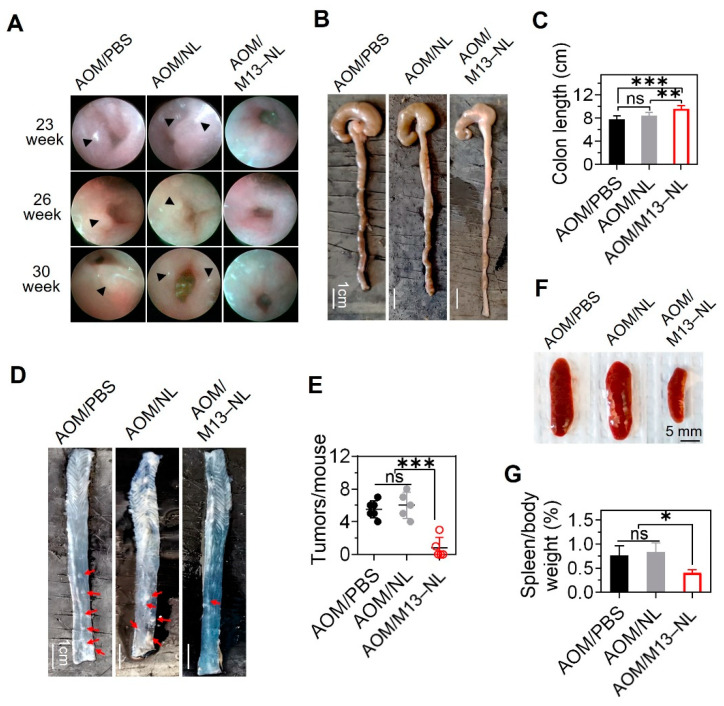
Cancer-preventing effect of M13–NL on AOM-exposed IL10^−/−^ mice. (**A**) Representative endoscopy results obtained at the indicated time points, black arrowheads indicate the location of tumors. (**B**) Representative images of the colons. (**C**) Comparison of colon lengths among different groups (*n* = 5 or 6). (**D**) Representative images of anatomical colon samples, red arrows indicate the location of tumors. (**E**) Comparison of the total number of tumors (*n* = 5 or 6). (**F**) Representative images of spleens from each group. (**G**) Assessment of the spleen-to-body weight ratio (*n* = 5). (ns, not significant; * *p* < 0.05, ** *p* < 0.01, and *** *p* < 0.001).

We assessed the long-term anti-tumorigenesis effect of M13–NL in the intestinal tract of IL10^−/−^ mice via colonoscopy. We detected obvious colorectal tumors in mice of the AOM/PBS and AOM/NL groups from weeks 23 to 30, whereas no obvious colorectal tumor was detected in mice of the AOM/M13–NL group during the same period (Figure 4A). After mice were sacrificed at week 31, we found that the colon lengths were significantly increased in the AOM/M13–NL group compared to those in the other two groups (Figure 4B,C). All mice in the AOM/PBS and AOM/NL groups had four to eight colon tumors (Figure 4D,E), whereas 60% of the M13–NL-treated mice (three out of five) had no colon tumor, and the rest, 40% (two out of five), had only one to three colon tumors; thus, the tumor burden was significantly lower in the M13–NL group than that in the AOM/PBS and AOM/NL groups (Figure 4E). Compared with the AOM/M13–NL group, the IL10^−/−^ mice of the AOM/PBS and AOM/NL groups showed clear signs of inflammation, including enlarged spleens and significantly higher spleen-to-body weight ratios (Figure 4F,G). 

### 3.4. Oral Administration of M13–NL Regulates Colonic Microbiota in the AOM-Exposed IL10^−/−^ Mice

**Figure 5:**
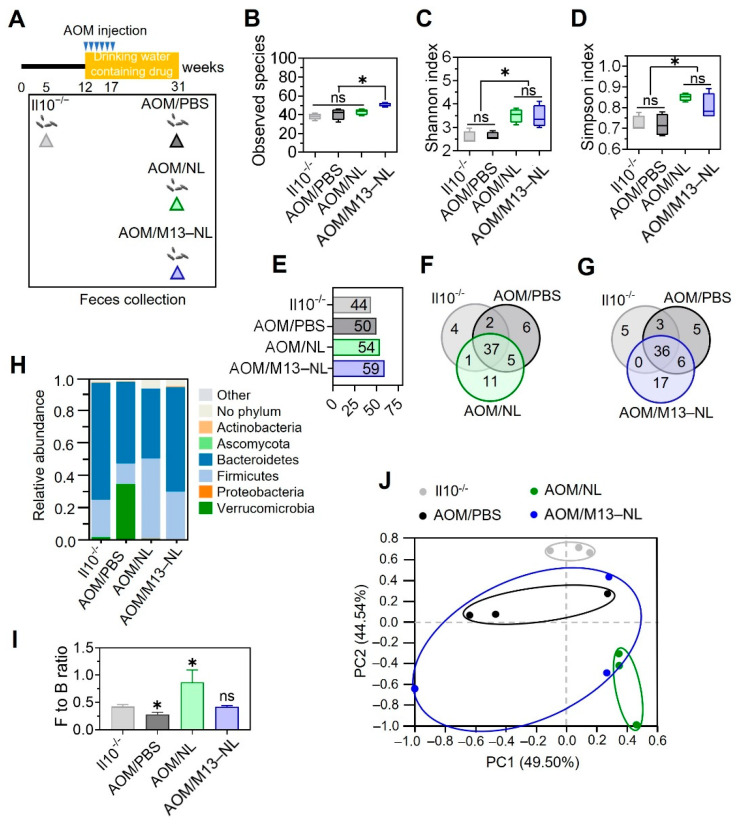
Remodeling effects of different formulations on the intestinal microbiota of AOM–exposed IL10^−/−^ mice. (**A**) Protocol for collecting feces at different time points from AOM–exposed IL10^−/−^ mice. Feces were collected from each group before AOM injection (on week 5, named IL10^−/−^ group) and before sacrifice (on week 31). (**B**) Observed species, (**C**) Shannon index, and (**D**) Simpson index calculated based IOTU levels of intestinal flora from different experimental groups (*n* = 3). (**E**) The total number of species in each group aIhe OTU level (>0.01%). (**F**) Venn diagrams of common and unique microbial species of mice among IL10^−/−^ vs. AOM/PBS vs. AOM/NL groups, and (**G**) IL10^−/−^ vs. AOM/PBS vs. AOM/M13–NL groups. (**H**) The relative abundance of the gut microbiome at the phylum level. (**I**) The Firmicutes to Bacteroidetes (F/B) ratio. (**J**) The PCA of each group at the phylum level (*n* = 3). Error bars represent one standard deviation (ns, not significant; * *p* < 0.05).

We analyzed the fecal microbiota compositions of IL10^−/−^ mice in each group (Figure 5A). The results indicated that the long-term oral administration of M13–NL significantly improved the α-diversity of the intestinal microbiota in AOM-exposed IL10^−/−^ mice, which exhibited higher relative abundances of intestinal microbiota compared to those of the other groups (Figure 5B). NL treatment, including that with both NL and M13–NL, also increased the α-diversity of the microbiota (Figure 5C,D), as indicated by the Shannon and Simpson indexes. For β-diversity (Figure 5H), the AOM/PBS group exhibited a reduction in the Firmicutes to Bacteroidetes (F/B) ratio, while the AOM/NL group exhibited an increase in the F/B ratio of the microbiota. In contrast, the M13–NL treatment group maintained the F/B ratio at the same low-inflammatory level seen in IL10^−/−^ mice before AOM injection (Figure 5I). PCA analysis of β-diversity demonstrated that AOM exposure significantly altered the microbiota compositions of IL10^−/−^ mice, moving them away from the pre-AOM injection state. There was no apparent distance or separation seen in the PCA results for the AOM exposure groups exposed to different treatments (Figure 5J), suggesting that M13–NL treatment had only a subtle impact on the β-diversity of gut microbiota. 

### 3.5. Oral Administration of M13–NL Increased Intratumoral Microbiota Species in the AOM-Exposed IL10^−/−^ Mice

**Figure 6:**
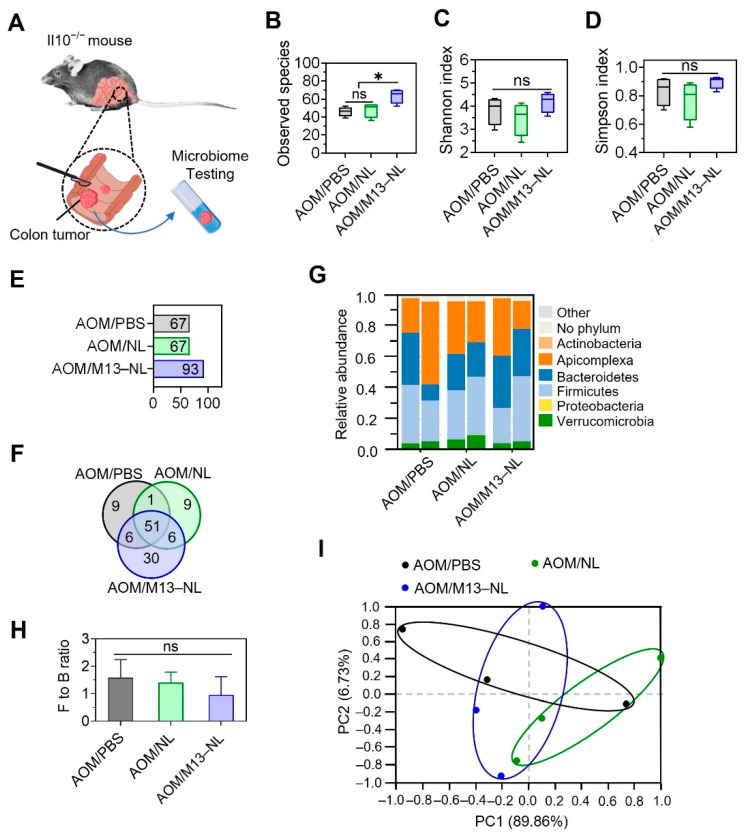
Effects of different formulations on the microbiota of tumor tissues in AOM–exposed IL10^−/−^ mice. (**A**) Schematic representation of the protocol for collecting intestinal tumor tissues from AOM–exposed IL10^−/−^ mice for microbiome testing. The intestinal tumor tissues of mice from AOM/PBS, AOM/NL, and AOM/M13–NL groups were carefully separated using a scalpel and placed in individual tubes containing DNA stabilization buffer for microbiome testing. (**B**) The observed species, (**C**) Shannon index, and (**D**) Simpson index calculated Ied on OTU levels of intestinal flora from different experimental group(*n* = 3). (**E**) The total number of species in eacIroup at the OTU level (>0.01%). (**F**) A Venn diagram of common and unique microbial species among the three groups. (**G**) The relative abundance of tumor microbiome at the phylum level. (**H**) ThFirmicutes to Bacteroidetes (F/B) ratio. (**I**) The PCA of each group at the phylum level (*n* = 3). Error bars represent one standard deviation (ns, not significant; * *p* < 0.05).

We also detected the microbiota composition inside colon tumors carefully dissected from the mice of each group (Figure 6A). We found that the number of microbial species in colon tumors was significantly higher in the AOM/M13–NL group than that in the AOM/PBS or AOM/NL groups (Figure 6B), but the α-diversity of microbiota in intestinal tumor tissues was similar among the three groups (Figure 6C,D). Concerning β-diversity (Figure 6G), although the AOM/NL treatment PBS groups showed a decreasing trend in the F/B ratio, this change was not significant (Figure 6H). The PCA of β-diversity also showed that there was no apparent distinction in intratumoral microbiota composition among the treatment groups (Figure 6I). These data indicate that the M13–NL treatment did not affect the composition of intratumoral microbiota, but did increase the number of microbial species inside the colon tumors.

## 4. Discussion

The application of nano-liposomes (NL) assembled using lipids extracted from ginger-derived nanoparticle band 2 (GDNPs-2) offers several advantages over that using the original GDNPs-2, including improved ease of storage and enhanced reproducibility. The latter carried various biomolecules, including proteins, microRNAs, and secondary metabolites such as gingerols and shogaols. These components could vary in concentration and affect the quality and stability of the nanoparticles [14]. GDNPs-2 offer colon-targeting properties, and the extracted lipids maintain this function while being more precisely defined. The reassembled NLs also lack toxicity, exhibit low immunogenicity, and are easy to mass-produce. In previous studies, we confirmed that reassembled lipid nanoparticles encapsulating M13 exhibit good anti-inflammatory activity in the intestinal tract of mice with UC and can promote intestinal wound healing upon oral administration [15]. The current study focused on examining the cancer-preventing ability of M13–NL.

The development of cancer frIm colitic tissue is a complex and lengthy process. Cancerous cells can exhibit unique features that can hamper the use of nanoparticles in cancer treatment [26]. A main challenge is the need to ensure that the nanoparticles can be internalized by and deliver their payload within cancer cells without being repelled or eliminated [27]. Our in vitro cell uptake assay provides a proof-of-principle evidence suggesting that NL experiences ongoing uptake by cancerous epithelial cells. In a prior study [15], we successfully verified the efficient cellular uptake of drug-loaded nano-liposomes (NL) across various cell lines. Armed with this valuable insight, we confidently assert that both NL and drug-loaded NL are effectively internalized by cancerous epithelial cells.

The AOM-exposed IL10^−/−^ mouse model is a valuable preclinical tool for studying the development of CAC and testing potential therapies [17]. This model effectively mimics crucial features of CAC observed in humans, including chronic inflammation, aberrant crypt foci, dysplasia, and invasive adenocarcinomas. The IL10^−/−^ colitis mouse model closely resembles that of individuals with inflammatory bowel disease (IBD), enhancing the model’s relevance to human disease [28]. These mice spontaneously develop chronic inflammation and cancer, similarly to humans with IBD, and thus provide a suitable platform for assessing the potential preventive effectiveness of CAC drug formulations in IBD patients. 

In our previous study, we demonstrated that encapsulating NL (nano-liposomes) significantly enhanced the cytotoxicity of M13 in vitro and improved its anti-cancer effectiveness against AOM/DSS induced colon cancer [29]. To minimize the use of animals in long-term studies, we proceeded directly to examine the potential of M13–NL in protecting IL10^−/−^ mice exposed to AOM from developing CAC. Research indicated that IL10^−/−^ mice experiencing spontaneous inflammation often develop splenomegaly [30]. Here, we observed that the prolonged oral administration of M13–NL significantly reduced splenomegaly in these mice. Our results suggest that M13–NL treatment is safe, as it does not trigger immunogenicity or result in noticeable systemic toxicity.

Additionally, M13–NL treatment enhanced the composition of the intestinal microbiota, bringing it closer to that of low-inflammation IL10^−/−^ mice, and modestly increased the presence of microbial species in intestinal tumors. The treatment with M13–NL also had an impact on the F/B ratio, moving it towards that observed in low-inflammation IL10^−/−^ mice. Study had suggested that a higher F/B ratio may be associated with low-grade inflammation, increasing the risk of the development and progression of certain types of cancer [31]. This modulation of the F/B ratio potentially played a role in preventing the development of colitis-associated cancer [32]. Changes in the F/B ratio have the potential to influence the colonic microenvironment through various mechanisms, including the alteration of metabolite production, modulation of the immune system, and restoration of gut barrier functions [32]. However, further investigation is needed to better understand the complex interplay between the gut microbiome and host health.

## 5. Conclusions

This study aimed to explore the ability of the long-term oral administration of M13–NL to prevent the development of CAC in IL10^−/−^ mice exposed to AOM. Our findings demonstrate that M13–NL has the potential to alleviate spontaneous inflammatory responses, improve the composition of the intestinal microbiota, and inhibit CAC tumorigenesis without causing noticeable systemic toxicity. These results highlight the potential of M13–NL as a promising candidate for preventing colitis-associated cancer (CAC) in patients with inflammatory bowel disease (IBD). Further investigations are needed to validate these findings and assess the safety and efficacy of M13–NL through human clinical trials.

## Data Availability

The data underlying this article will be shared upon reasonable request to the corresponding author.

## Supplementary Materials

The following supporting information can be downloaded at: https://www.mdpi.com/article/10.3390/pharmaceutics15092331/s1, Figure S1: Size and PDI characterization of assembled NL and M13-NL.
