# Supplementary material for: Prevention of Colitis-Associated Cancer via Oral Administration of M13-Loaded Lipid Nanoparticles
Source: Pharmaceutics. 2023 Sep 16;15(9):2331. doi: 10.3390/pharmaceutics15092331 (PMC10534593; doi:10.3390/pharmaceutics15092331)
Supplement: Supplementary file 1 [file pharmaceutics-15-02331-s001.zip › pharmaceutics-2568219-supplementary.pdf]

Supplementary Information

Supplementary figures

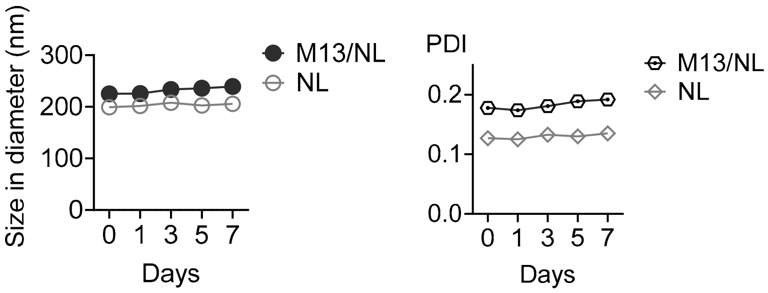

Figure S1. Size and PDI characterization of assembled NL and M13-NL.
